# *GYG1*: A distal myopathy with polyglucosan bodies

**DOI:** 10.1002/jmd2.12129

**Published:** 2020-05-13

**Authors:** Stefan Nicolau, Jennifer A. Tracy, David J. Pisapia, Kurenai Tanji, Margherita Milone

**Affiliations:** ^1^ Department of Neurology Mayo Clinic Rochester Minnesota USA; ^2^ Department of Pathology Weill Cornell Medical Center New York New York USA; ^3^ Department of Pathology and Cell Biology Columbia University Medical Center New York New York USA

**Keywords:** distal myopathy, glycogen storage disease, glycogenin‐1, polyglucosan body

## Abstract

Mutations in glycogenin‐1 (*GYG1*) cause an adult‐onset polyglucosan body myopathy. We report here a patient presenting with late‐onset distal myopathy. We wish to highlight this rare clinical phenotype of *GYG1*‐related myopathy and the histological clues leading to its diagnosis.

1

**Synopsis**

Mutations in glycogenin‐1 (*GYG1*) may cause an adult‐onset distal polyglucosan body myopathy.

A 68‐year‐old woman presented with a 10‐year history of distal myopathy predominantly affecting finger extensors and intrinsic hand muscles, with lesser weakness of proximal muscles. Electromyography showed myopathic findings. The creatine kinase level was normal. A muscle biopsy revealed a polyglucosan body myopathy (PGBM, Figure 1). Echocardiography was unremarkable. Genetic testing identified two pathogenic variants in *GYG1*: c.143+3G>C and c.819T>A, p.Tyr273Ter.

**FIGURE 1:**
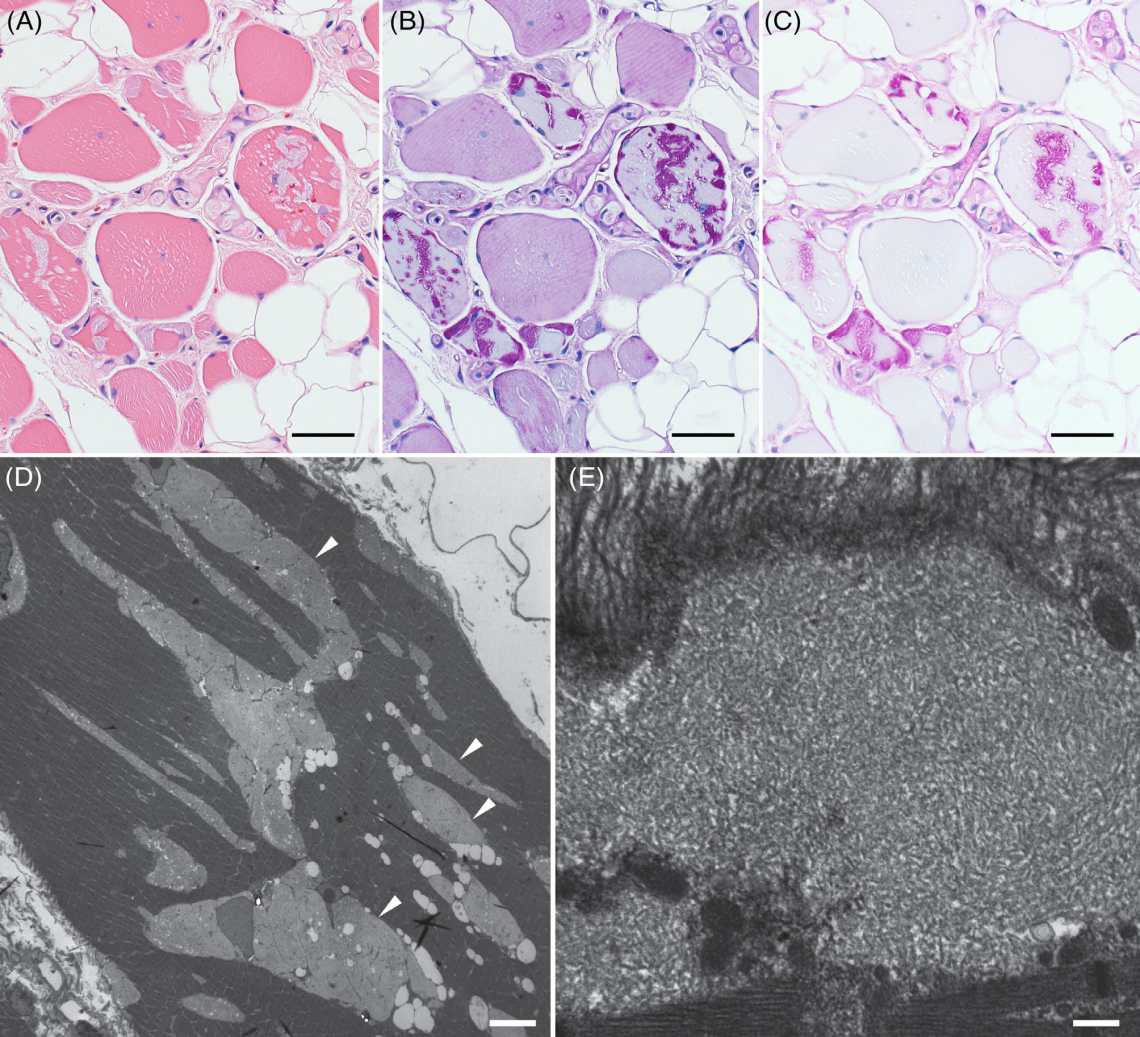
Deltoid biopsy demonstrating polyglucosan bodies. Haematoxylin and eosin preparation, A, showing large violaceous inclusions containing periodic acid‐Schiff‐positive material; B, resisting α‐amylase treatment; C, suggesting polyglucosan accumulation. Normal glycogen is depleted in fibres with polyglucosan bodies. Electron micrographs showing filamentous structures consistent with polyglucosan (arrowheads, D,E). Scale bar A to C, 50 μm; D, 10 μm; E, 400 nm

Polyglucosan is a polysaccharide with a less branched structure than glycogen and which aggregates into polyglucosan bodies. PGBM can stem from mutations in *GBE1*, *GYG1*, or *RBCK1*. Polyglucosan bodies in muscle can also occur in phosphofructokinase deficiency, Lafora disease or PRKAG2‐cardiomyopathy.^1^ *GYG1* encodes glycogenin‐1, an enzyme whose autoglycosylation is one of the initial steps of glycogen synthesis. Mutations in *GYG1* cause glycogen storage disease (GSD) XV. c.143+3G>C is the most common pathogenic variant in *GYG1* and causes skipping of exon 2, leading to a frameshift and nonsense‐mediated decay.^2^, ^3^ The c.819T>A variant is novel and located between the catalytic and C‐terminal glycogen synthase‐binding domains. Truncating variants in the catalytic domain result in reduced or absent protein expression, while those in the C‐terminal region result in a protein with reduced glycosylation.^1^, ^2^, ^3^ Like our patient, those carrying the latter variants also presented with distal weakness.^2^, ^3^

Age of onset of GSD XV varies widely, ranging from childhood to the 70s.^2^, ^3^ The disorder most commonly presents with slowly progressive proximal weakness, but scapuloperoneal and distal myopathy have also rarely been reported.^3^ Some patients exhibit significantly asymmetric weakness. The distal myopathy phenotype is associated with an older age of onset (40s‐70s).^3^ Unlike several other GSDs, exercise intolerance and rhabdomyolysis are not prominent features. Creatine kinase levels are normal or mildly elevated. It has been suggested that polyglucosan bodies in GSD XV are more easily digested by α‐amylase, potentially offering a clue to the diagnosis.^2^ *GYG1* is also expressed in cardiac muscle, and cardiomyopathy has occasionally been reported in GSD XV, with or without skeletal muscle involvement.

## 2 CONFLICT OF INTEREST

S. N., J. T., D. P., and K. T. declare that they have no conflict of interest. M. M. receives compensation as associate editor of Neurology Genetics.

## AUTHOR CONTRIBUTIONS

Stefan Nicolau: Data collection and writing of the first draft of the manuscript. Jennifer A. Tracy, David J. Pisapia, Kurenai Tanji: Biopsy interpretation and critical review of the manuscript. Margherita Milone: Data collection, supervision and critical review of the manuscript; serves as guarantor for this article, accepts full responsibility for the work and the conduct of the study, had access to the data, and controlled the decision to publish.

## References

[1] Hedberg‐Oldfors C , Oldfors A . Polyglucosan storage myopathies. Mol Aspects Med. 2015;46:85‐100. 26278982 10.1016/j.mam.2015.08.006

[2] Malfatti E , Nilsson J , Hedberg‐Oldfors C , et al. A new muscle glycogen storage disease associated with glycogenin‐1 deficiency. Ann Neurol. 2014;76(6):891‐898. 25272951 10.1002/ana.24284 PMC4348070

[3] Ben Yaou R , Hubert A , Nelson I , et al. Clinical heterogeneity and phenotype/genotype findings in 5 families with GYG1 deficiency. Neurol Genet. 2017;3(6):e208. 29264399 10.1212/NXG.0000000000000208 PMC5735306

